# Identification of CVID Patients With Defects in Immune Repertoire Formation or Specification

**DOI:** 10.3389/fimmu.2018.02545

**Published:** 2018-11-23

**Authors:** Pauline A. van Schouwenburg, Hanna IJspeert, Ingrid Pico-Knijnenburg, Virgil A. S. H. Dalm, P. Martin van Hagen, David van Zessen, Andrew P. Stubbs, Smita Y. Patel, Mirjam van der Burg

**Affiliations:** ^1^Department of Immunology, Erasmus MC University Medical Center, Rotterdam, Netherlands; ^2^Division of Clinical Immunology, Department of Internal Medicine, Erasmus MC University Medical Center, Rotterdam, Netherlands; ^3^Clinical Bioinformatics Unit, Department of Pathology, Erasmus MC University Medical Center, Rotterdam, Netherlands; ^4^Nuffield Department of Clinical Medicine and Oxford NIHR Biomedical Research Centre, University of Oxford, Oxford, United Kingdom

**Keywords:** common variable immune deficiency disorder, B-cell receptor repertoire, repertoire formation, repertoire specification, germinal center, somatic hypermutation

## Abstract

Common variable immune deficiency disorder (CVID) is the most clinically relevant cause of antibody failure. It is a highly heterogeneous disease with different underlying etiologies. CVID has been associated with a quantitative B cell defect, however, little is known about the quality of B cells present. Here, we studied the naïve and antigen selected B-cell receptor (BCR) repertoire in 33 CVID patients using next generation sequencing, to investigate B cells quality. Analysis for each individual patient revealed whether they have a defect in immune repertoire formation [V(D)J recombination] or specification (somatic hypermutation, subclass distribution, or selection). The naïve BCR repertoire was normal in most of the patients, although alterations in repertoire diversity and the junctions were found in a limited number of patients indicating possible defects in early B-cell development or V(D)J recombination in these patients. In contrast, major differences were found in the antigen selected BCR repertoire. Here, most patients (15/17) showed a reduced frequency of somatic hypermutation (SHM), changes in subclass distribution and/or minor alterations in antigen selection. Together these data show that in our CVID cohort only a small number of patients have a defect in formation of the naïve BCR repertoire, whereas the clear majority of patients have disturbances in their antigen selected repertoire, suggesting a defect in repertoire specification in the germinal centers of these patients. This highlights that CVID patients not only have a quantitative B cell defect, but that also the quality of, especially post germinal center B cells, is impaired.

## Introduction

Common variable immune deficiency disorder (CVID) is the most frequent, clinically relevant antibody deficiency. The most prominent features of CVID include increased risk of (recurrent) infections, low serum IgG in combination with low serum IgM and/or IgA and poor antigen specific antibody responses. In addition, non-infection complications such as granulomas, auto-immunity or malignancies are found in part of the patients (1). According to the ESID diagnostic criteria of CVID, any defined cause of antibody deficiency must be excluded, leaving a highly heterogeneous patient group in terms of clinical features and underlying disease etiologies. The majority of CVID cases is thought to be polygenic in nature (2, 3), although monogenic defects have been described in an increasing number of patients (4–6). CVID is associated with a defect in B-cell development, but the affected stage differs between patients (7).

B cells develop in the bone marrow, where they undergo V(D)J recombination to produce a unique B-cell receptor (BCR). In each precursor B cell, one variable (V) gene is combined with one diversity (D) and one joining (J) gene to create the immunoglobulin heavy chain (IGH) encoding gene. The high number of combinations of V, D, and J genes together with the addition of non-templated (N)-nucleotides and the deletion of nucleotides in the junctional regions allows for the generation of a great variety of BCRs. After successfully completing V(D)J recombination on the IGH locus, and rearranging the light chain (IGL) the formed BCR is tested for auto-reactivity and if needed auto-reactive B-cells undergo receptor editing or apoptosis while non-auto-reactive B-cells migrate to the periphery and differentiate into naïve B-cells. The total set of BCRs of naïve B-cells is referred to as the naïve BCR repertoire which is the result of repertoire formation in the bone marrow.

In the periphery, naïve B cells become activated upon antigen recognition. Upon B-T cell interaction, activated B cells migrate to the dark zone of the germinal center (GC) where they undergo rapid proliferation and somatic hyper mutation (SHM). During SHM, mutations are introduced in the BCR encoding genes allowing further specification of the receptor. SHM is initiated by AID, which deaminates a cytosine into a uracil (U) creating U lesions mainly in RGYW/WRCY motifs (R = Purine, Y = pyrimidine, W = A or T) which can be resolved via three main pathways. First, during error prone base excision repair (BER) the U is removed and replaced by error-prone translesion synthesis (TLS) polymerases leading to transitions or transversions at C/G locations (8, 9). Second, during error prone mismatch repair (MMR) the U and multiple bases surrounding the mismatch are excised (10–12). Then, the error prone polymerase pol eta fills the single stand gap, preferentially making errors at AT locations (TW/WA motifs). Finally, in absence of repair, the U is recognized as a T during replication leading to transitions at G/C locations.

Subsequently, B cells migrate to the light zone of the GC where they undergo antigen selection and class switch recombination (CSR). During antigen selection, B cells with SHMs leading to a BCR with increased affinity get selected for survival, while cells with affinity decreasing mutations undergo apoptosis (13). During CSR the constant domain of the BCR encoding gene is replaced which changes the effector function of the antibodies produced (13). The resulting set of BCR, developed upon specification of the repertoire in the GC is defined as the antigen selected repertoire.

In previous studies it has been shown that CVID patients often have a quantitative defect in B cell development, however, less is known about the quality of the developed B cells (7, 14, 15). We have previously analyzed the naïve BCR repertoire in a cohort of 18 CVID patients, and found that the majority of patients had a normal naïve BCR repertoire with respect to gene segment usage, composition of the complementarity determining region 3 (CDR3) and receptor diversity suggesting normal repertoire formation (16). In contrast, Roskin et al. recently reported alterations in VDJ reagerangement, CDR3 formation, diversity and SHM upon the analysis of the BCR repertoire in CVID patients suggesting defects in repertoire formation and specification in their cohort (17). To get a more detailed view to which extent repertoire formation and specification is affected in individual CVID patients, we now studied both the naïve BCR repertoire and antigen selected BCR repertoire on sorted or selected B-cells in a group of 33 CVID patients using next generation sequencing (18). In contrast to previous studies, we focus on deviations per patient to be able to identify processes affected in individual patients instead of comparing CVID patients as a group to controls. We performed detailed analysis on all the parameters associated with auto-immunity and parameters associated with repertoire formation [i.e., V(D)J recombination] and specification (SHM, CSR, and antigen selection). In parameters of the naïve repertoire, only minor differences were observed in a minority of patients. In contrast, our data showed major skewing in the antigen selected repertoire including SHM frequencies and IGH subclass distribution in the majority of CVID patients, in line with defects in the GC. Together this suggest that repertoire formation is intact in the majority of CVID patients, while repertoire specification is impaired in the majority of patients.

## Materials and methods

### Healthy donors and patients

Peripheral blood samples were collected from 33 HCs and 33 CVID patients (age 6 −79 y). Patients were recruited to the study “Primary Immune Deficiencies: immunological and genetic background in relation to clinical complications” (ErasmusMC METC MEC-2013-026/NL40331.078.12) or the study “The genetic and functional characterization of patients with Primary Immune Deficiencies, Infections and Inflammatory conditions” (South Hampshire Research Ethics Committee, 12/SC/0044). The study was reviewed and approved by the above-mentioned ethics committees and all subjects gave written informed consent in accordance with the Declaration of Helsinki. Data of the naïve repertoire of 10 HC's and 15 CVID patients were previously published (16). From this previous study we excluded three CVID patients in which a (possible) genetic defect has been identified. An overview of the included patients, B-cell and T-cell subsets and antibody levels at diagnosis can be found in Supplementary Table 1.

### Repertoire sequencing

Peripheral blood mononuclear cells (PBMCs) were isolated using Ficoll. For naïve BCR repertoire DNA was isolated from sorted naïve B-cells (CD27–, IgD+) using direct lysis (19). Next, 100 ng (or less when insufficient DNA was available) of DNA was amplified using VH1-6 FR1 forward primers (BIOMED-2) and JH consensus reverse primers (20). For analysis of the antigen selected BCR repertoire mRNA was isolated from total PBMCs using the Gen-Elute Mammalian total RNA miniprep kit from Sigma Aldrich (St. Louis, MO). cDNA was created from 2 ug RNA [Superscript II reverse transcriptase kit Invitrogen (Paisley, UK)]. IGH transcripts were amplified in a multiplex PCR using forward VH1-6 FR1 (BIOMED-2) primers and a CgCH1 or IGHA reverse primer (20–22). All PCR products were purified and sequenced using Roche 454 sequencing as previously described (23). In short, PCR products were purified by gel extraction (Qiagen, Valencia, CA) and Agencourt AMPure XP beads (Beckman Coulter, Fullerton, CA). The PCR concentration was measured using the Quant-it Picogreen dsDNA assay (Invitrogen, Carlsbad, CA) and the purified PCR products were sequenced on the 454 GS junior instrument using the Lib-A or Lib-A V2 kits.

### Immune repertoire data analysis

Sequences were demultiplexed based on their multiplex identifier sequence and trimmed from both sides (antigen selected BCR repertoire: 40 nt; naïve BCR repertoire: 40 nt) (18). Fasta files were uploaded in IMGT/High-V-Quest (version 1.5.6 for the antigen selected BCR repertoire) (24), and subsequently the IMGT output files were analyzed using ARGalaxy (18). For analysis of the naïve BCR repertoire a single sequence per clone (based on V gene, and CDR3 nucleotide sequence) was selected for analysis. Replicates in which this resulted in < 100 sequences were excluded. For the antigen selected BCR repertoire only sequences present two or more times (based on CDR1-CDR3 nucleotide sequence) were included once in the analysis. In addition, incomplete sequences or sequences containing an ambiguous “n” base were excluded. Samples containing a low number of reads for either IGHA or IGHG after filtering (< 45) or patients samples with increased clonal relation as compared to HC's (Supplementary Table 2) were excluded. The age, presence of autoimmunity, and number of sequences after filtering (Supplementary Table 2), and the transition tables (Supplementary Table 3) can be found in the Supplementary Information. To determine the selection strength of the CDR and FR regions the BASELINe tool was used (selection statistics: focused, SHM targeting model: human Tri-nucleotide, custom boundaries: 25:26:38:55:65:104:-) (25, 26). Clonal relation between sequences was determined using Change-O using the nucleotide hamming distance substitution model with a complete distance of maximal three. For clonal assignment the first genes were used, and the distances were not normalized. In case of asymmetric distances, the minimal distance was used (27).

### Data visualization and statistics

Dot plots and statistics were performed using Graphpad Prism V7.0. PCA analysis and heatmaps (heatmap2; gplots package) were made using R.

## Results

To investigate whether CVID patients have alterations in their BCR repertoire that might indicate defects in repertoire formation or specification, we analyzed the naïve and the antigen selected BCR in CVID patients and age-matched healthy controls (HC's). To analyse the naïve BCR repertoire we sorted naïve B cells from 30 CVID patients and 16 HC's and amplified and sequenced the *IGH* gene. We performed 4–6 replicate PCR's which allowed us to determine the number of unique rearrangements that overlap between independent PCRs from the same individual (co-incidences) and calculate repertoire diversity (28). In two patients the BCR encoding region could not be amplified, probably due to low DNA levels, and these were excluded. For one patient only two replicates resulted in >100 reads and this patient was excluded for the calculation of repertoire diversity (Supplementary Table 2). To analyse the antigen selected repertoire, we amplified and sequenced IGHG and IGHA transcripts from peripheral blood mononuclear cells PBMCs from 22 CVID patients including 11 patients of which we also analyzed the naïve repertoire. Results were compared to previously obtained aged-matched HC's (29) From 18 patients >45 unique reads from IGHG and/or IGHA transcripts were obtained. IGHA and/or IGHG data from five patients were excluded based on increased clonal relation of the data (Supplementary Table 2) (27). In total the antigen selected repertoire of 16 patients was analyzed [IGHG and IGHA (*n* = 9); IGHG only *n* = 6 and IGHA only *n* = 1; Supplementary Table 2].

### Some CVID patients have reduced diversity of the naïve BCR repertoire and small alterations in the junctional regions

**Figure 1:**
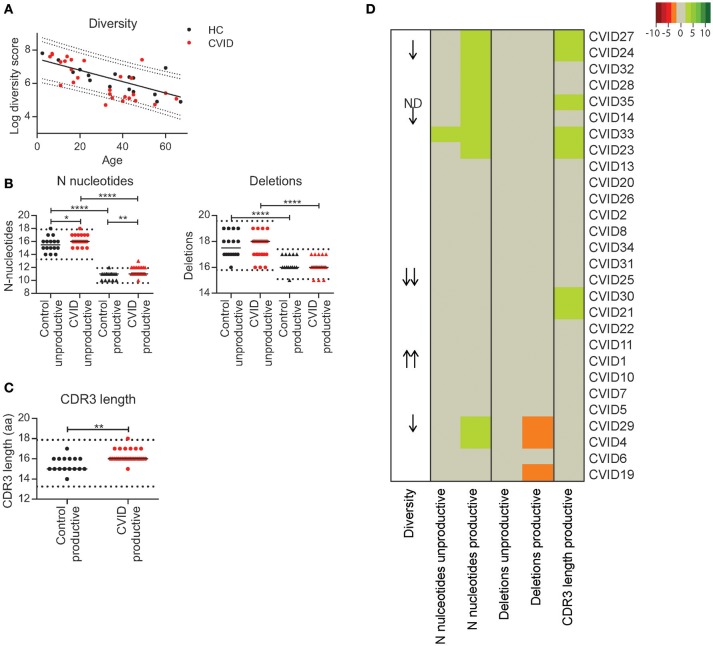
Repertoire diversity and junction characteristics in CVID patients. **(A)** Repertoire diversity in HC's (black) and CVID patients (red). A trendline representing the correlation between age and repertoire diversity in HC's is added. The dotted lines represent the 90 and 95% prediction bands based on the HC population. **(B)** The number of N-nucleotides and deletions in the junctions of productive and unproductive rearrangements from HC's and CVID patients and **(C)** the median CDR3 length in productive rearrangements. The dotted lines represent the mean ±2 standard deviations (st dev) of the HC. **(D)** Heatmap showing the junction characteristics of each patient. Values below 2 st dev from the mean of the HC samples are indicating by orange, while larger deviations from the mean are indicated by darker shades of red. Values above 2 st dev from the mean are indicated with green with darker green representing increased deviation from the HC population. Patients were clustered based on junction characteristics. Patients with a reduced or increased repertoire diversity are indicated by arrows (One arrow: within the 90–95% prediction bands; two arrows: outside of the 95% prediction bands). Statistical significance is performed using a Mann Whitney-test and indicated using *P < 0.05, **P < 0.01 and ****P < 0.0001.

To determine if individual CVID patients have a possible defect in immune repertoire formation [V(D)J recombination] we analyzed repertoire diversity and junction characteristics in sorted naïve B-cells. In the HC's we found that the diversity of the naïve BCR repertoire decreases with age (Figure 1A). For the majority of CVID patients, the naïve repertoire diversity was within the 90% prediction bands of the HC's. However, for three patients the diversity was below the lower 90% prediction band and for one patient below the 95% prediction band. Surprisingly, one patient had a diversity above the upper 95% prediction band (Figure 1A). In the junctions of the CVID patients we found normal numbers of N-nucleotides and deletions in the unproductive IGH rearrangements in all except for one patient in which the number of N-nucleotides was marginally increased (CVID33, Figure 1B). Unproductive rearrangements reflect the rearrangements formed during V(D)J recombination that are not expressed and therefore not selected.

In the productive IGH rearrangements the number of N-nucleotides was above the normal range in 10 CVID patients. Although this increase was minor, it was often associated with a longer CDR3 length (Figures 1B,C). In addition, three patients had a reduced number of deletions in their productive rearrangements. In all except one (CVID33) patient, these minor alterations in the junctions of productive rearrangements were not associated with alterations in the junctions of unproductive rearrangements and therefore it is unlikely the result of defects during V(D)J recombination. Interestingly, in three patients these alterations in the productive junctions did coincide with reduced BCR repertoire diversity (CVID 14, CVID24 CVID29).

Based on these data, we concluded that our cohort does not include CVID patients with major defects in the processing phase of V(D)J recombination. However, the patients with reduced naïve repertoire diversity might have a mild defect in initiation of V(D)J recombination or a different defect in precursor B-cell development. The alterations in the junctions of the productive rearrangements could indicate minor disturbances in B-cell development downstream of the V(D)J recombination process.

### Minor skewing in SHM patterns in CVID patients

**Figure 2:**
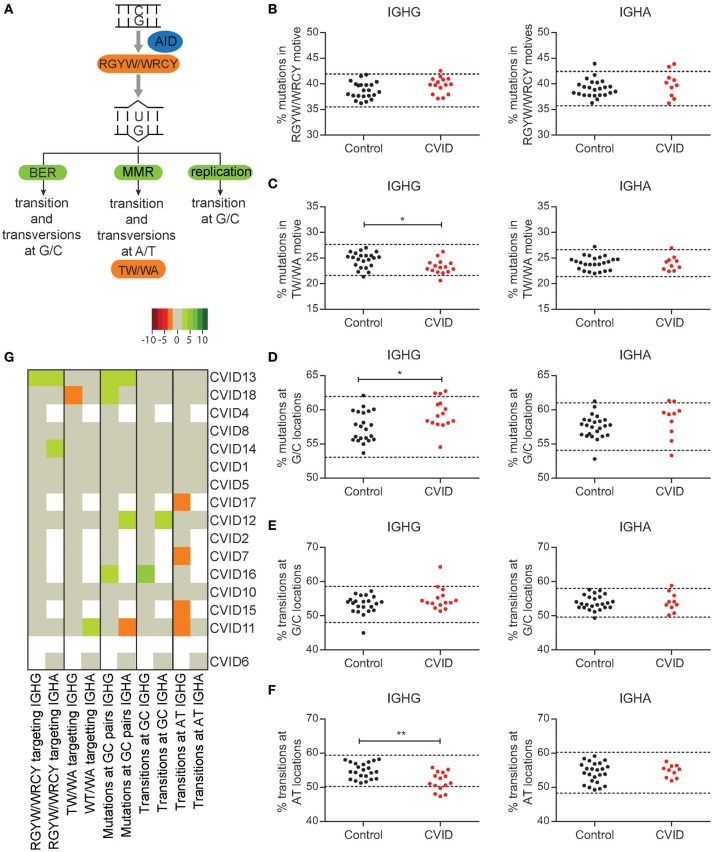
Minor skewing in SHM patterns are found in a subgroup of CVID patients. **(A)** A schematic overview of the different pathways by which AID induced U lesions can be repaired and the mutation patterns they are linked to. The percentages of mutations located in RGYW/WRCY **(B)** and TW/WA **(C)** motives in IGHA and IGHG rearrangements of HC's and CVID patients. **(D)** Percentage of mutations located at G/C locations. The percentage of mutations located at G/C **(E)** or A/T **(F)** locations that are transitions. The dotted lines represent the mean ±2 standard deviations (st dev) of the HC. **(G)** Heatmap showing the different aspects of SHM patterns per patient. Values < 2 st dev below the mean are depicted in increasingly darker shades of red, while values >2 st dev above the mean are depicted in increasingly darker shades of green. Patients (except CVID6) are clustered based on all data included in the heatmap. Dotted lines in graphs B-F represent the mean plus and minus 2 st dev of the HC. Statistical significance is performed using a Mann Whitney-test and indicated using *P < 0.05 and **P < 0.001.

To investigate if there are CVID patients in our cohort with a defect in the molecular mechanism of SHM we analyzed the percentage of mutations in AID (RGYW/WRCY) and pol eta (WA/TW) motifs (Figures 2A–C), the percentage of mutations at GC location (Figure 2D), and the percentage of transition mutations at GC (Figure 2E) or A/T locations (Figure 2F). We found that seven patients have normal SHM patterns, suggesting a normal distribution between the repair pathways (Figure 2G). In four patients, minor skewing was observed in one subclass, but the same skewing could not be found in the other subclass making a defect in the molecular mechanism of SHM unlikely (CVID11, CVID12, CVID14, CVID18). CVID13 had skewing that could match reduced efficiency of MMR. Of the remaining four patients three had increased transitions at AT locations (CVID7, CVID15, and CVID17) and one patient had increased mutations at CG locations and increased transitions at GC locations (CVID16). On a population basis, these minor alterations lead to reduced pol eta targeting, increased mutations at GC locations, and decreased number of transitions at A/T locations in IGHG transcripts of CVID patients. Interestingly, no significant skewing was found in IGHA transcripts. Together these data indicate that the majority of patients do not have a defect in the molecular mechanism of SHM. Some slight skewing in SHM patterns were found in some patients which could indicate minor defects in pathways involved in SHM. However, these defects are unlikely to be disease causing in these patients.

### The majority of CVID patients have reduced SHM levels and/or reduced usage of distal constant domains

**Figure 3:**
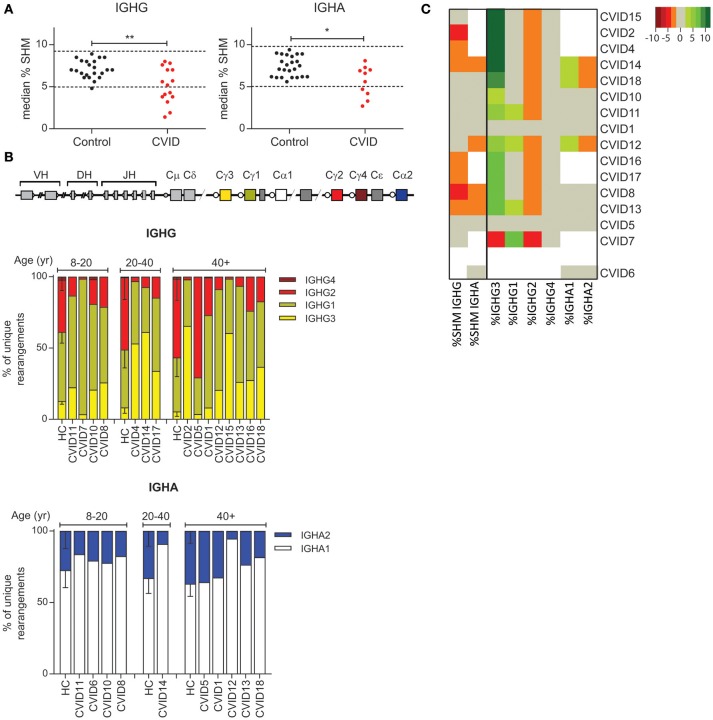
CVID patients often have reduced levels of SHM and reduced usage of more distal constant domains. **(A)** Median SHM levels in IGHG and IGHA transcripts from HC's and CVID patients. The dotted line indicates the mean ±2 st dev of the control samples. Statistical significance is performed using a Mann Whitney-test and indicated using *P < 0.05 and **P < 0.01. **(B)** Schematic overview of the IGH locus and subclass distribution in IGHA and IGHG transcripts of CVID patients and aged matched HC's. **(C)** Heatmap showing the levels of SHM and subclass distribution in each patient. Patients who deviate more than 2 st dev from the mean are depicted in increasingly darker shades of green (increased levels) or red (decreased levels). Patients (except CVID6) are clustered based on all heatmap data.

It has been shown that a subgroup of CVID patients has a defect in the GC (7). Since SHM and CSR take place in the GC, we used these parameters to identify patients with a possible GC defect. Our results show that eight out of 16 CVID patients have reduced SHM frequency in IGHG and/or IGHA rearrangements (Figure 3A) leading to a significant decrease in SHM levels in CVID patients as compared to HC's. Strikingly, the distribution of the IGHG subclasses was altered in 13 of the 15 patients in whom IGHG transcripts were analyzed (Figure 3B). In these patients the frequency of IGHG2 transcripts was reduced while the frequencies of IGHG3 or IGHG1 transcripts were often increased. The subclass distribution of IGHA transcripts was abnormal in three patients. Reduced SHM frequency always coincided with altered subclass distribution (Figure 3C). Together these data show that the majority of our CVID patients have reduced SHM and altered subclass distribution which might be indicative of a defect in the GC, or alternatively B-cell activation.

### CVID patients show minor alterations in GC selection

Next to SHM and CSR a third process taking place in the GC is antigen selection. CVID patients have an impaired vaccination response, which could be the effect of poor antigen selection and affinity maturation. A hallmark of antigen selection is an increased frequency of replacement (R) mutations relative to silent mutations (S) in the complementary determining region (CDR; R/S ratio). In contrast, the R/S ratio in the framework region (FR) is low as R mutations in the FR can cause BCR instability. In addition, we used Bayesian estimation of Antigen-driven SELectIoN (BASELINe) to calculate antigen selection in the CDR and FR regions (30).

**Figure 4:**
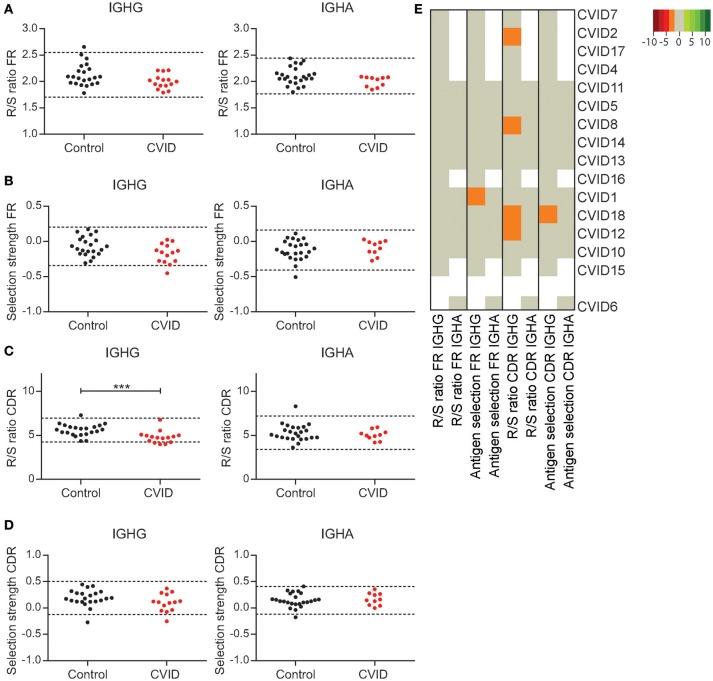
CVID patients show minor alterations in GC selection. The R/S ratio **(A)** and selection strength as analyzed by BASELINe **(B)** in the FR region of IGHA and IGHG transcripts. **(C)** The R/S ratio in the CDR regions of IGHA and IGHG transcripts. **(D)** The BASELINe selection strength in the CDR regions. Dotted lines in graphs **(A–D)** represent the mean plus and minus 2 st dev. Statistical significance is performed using a Mann Whitney-test and indicated using ***P < 0.0005. **(E)** Heatmap representing all outcomes for antigen selection per patient. Values below 2 st dev from the mean of the HC samples are indicating by orange, while larger deviations from the mean are indicated by darker shades of red. Values above 2 st dev from the mean are indicated with green with darker green representing increased deviation from the HC population. Patients are clustered based on all antigen selection parameters included.

In HC's both the R/S ratio and the BASELINe selection strength in the FR regions of IGHG and IGHA transcripts is stable [Figures 4A,B; (29)]. One CVID patient shows reduced antigen selection in the FR regions of IGHG rearrangements according to BASELINe, however this finding was not confirmed by an altered R/S ratio or alterations in the selection of IGHA transcripts. When analyzing the selection in the CDR regions, we found a reduced R/S ratio in the IGHG transcripts of four patients, while the BASELINe selection strength was reduced in one CVID patient (Figures 4C,D). However, these findings did not coincide (Figure 4E), suggesting that none of the CVID patients have major defects in antigen selection. Surprisingly, at the cohort level we found a significant reduced R/S ratio in the CDR of IGHG rearrangements of CVID patients. Together this suggests that although almost all of the CVID patients seem to have a defect in the GC, antigen selection was not impaired in the IGHG or IGHA transcripts of individual patients.

### CVID patients show reduced selection against repertoire features associated with auto-immunity

**Figure 5:**
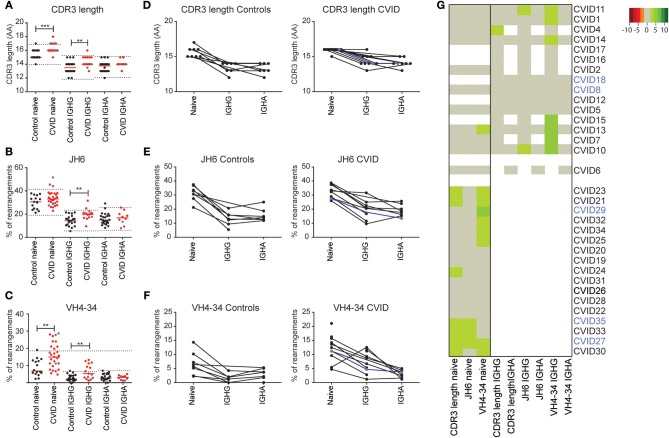
CVID patients have increased VH4-34 and JH6 usage and an increased CDR3 length. Mean CDR3 length **(A)** the percentage JH6 **(B)** and VH4-34 usage **(C)**, in naïve B-cells and IGHA and IGHG transcripts of antigen selected B-cells in all samples or samples of which paired data was available **(D–F)**. The dotted lines in **(A–C)** represent the mean ± the st dev of the controls. **(G)** Heatmap showing VH4-34 and JH6 usage and median CDR3 length for all patients. Values >2 st dev below the mean are depicted in increasingly darker shades or red, while values >2 st dev above the mean are depicted in increasingly darker shades of green. Patients are clustered based on the data included in the heatmap. CVID patients with autoimmunity are indicated in blue. Statistical significance is performed using a Mann Whitney-test and indicated using *P < 0.05, **P < 0.01 and ***P < 0.001.

Besides antigen selection, there is strong selection against features associated with auto-immunity in both the bone marrow and the GC. These features include a long CDR3 length and the usage of the intrinsically auto-reactive IGHV4-34 gene (29, 31). In the naïve BCR repertoire we found a marginal increase in CDR3 length in seven CVID patients (Figure 5A), which coincided with increased JH6 usage (the longest JH gene) in three patients (Figure 5B). In nine patients, the percentage of rearrangements using the IGHV4-34 gene was increased which was associated with auto-immunity in two patients (CVID27, CVID29; indicated in blue) (Figure 5C). The total group of CVID patients has a significant increase in CDR3 length and increased IGHV4-34 gene usage in their naïve BCR repertoire as compared to HC's (Figures 5A,B). In the antigen selected BCR repertoire, the CDR3 length of the IGHG and IGHA transcripts is shorter compared to the naïve B cells in both the HC's and CVID patients. However, in CVID patients the IGHG and the IGHA transcripts have a longer CDR3 compared to the HC's. We found one CVID patient with a minor increase in median CDR3 length in IGHG transcripts (CVID4) and two patients with increased JH6 usage (CVID10, CVID 11) (Figures 5A,B). More pronounced differences were found in the IGHV4-34 gene usage in CVID patients (Figures 5C,G) which showed increased usage of VH4-34 in IGHG but not IGHA transcripts of seven patients. However, again these auto-immune features were not associated with clinical auto-immunity in these patients.

### CVID patients have only minor alterations in the naïve BCR repertoire, while major skewing is found in the antigen selected BCR repertoire

**Figure 6:**
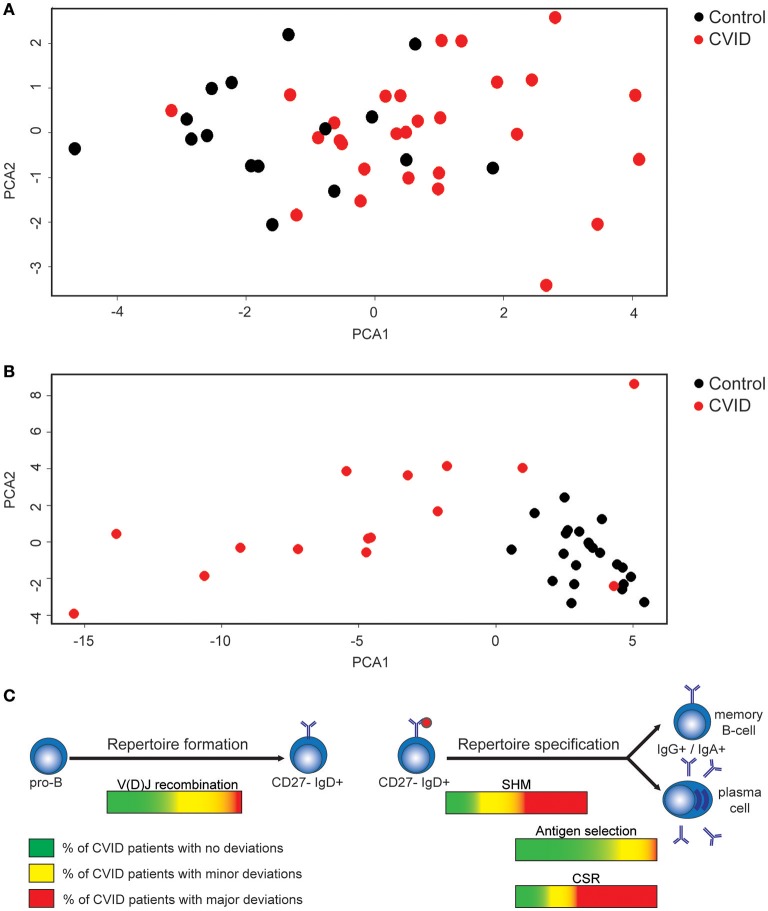
Minor alterations in the naïve BCR repertoire in a subgroup of CVID patients and alterations in the antigen selected IGHG repertoire in the majority of CVID patients. **(A)** PCA analysis of CVID patients and HC's based on all data extracted from the naïve BCR repertoire. **(B)** PCA analysis based on the antigen selected BCR repertoire of HC's and CVID patients. **(C)** Figure summarizing the processes during repertoire formation and specification that are disturbed in CVID patients.

We investigated if alterations found in the naïve BCR repertoire of CVID patients (Figures 1, 5) coincided in the same patients. Therefore, we created a heatmap representing all data presented above (Supplementary Figure 1). Data shows that 12 CVID patients have a completely normal naïve BCR repertoire while in the remaining 16 patients, minor alterations in the diversity, junctions or auto-immune features were identified. Since all differences found in the naïve BCR repertoire are minor, principal component analysis (PCA) showed the majority of CVID patients clustering together with the HC's (Figure 6A; Supplementary Figure 2A).

In the antigen selected BCR repertoire more pronounced differences were found (Supplementary Figure 3). Only in two patients (CVID5 and CVID6) all parameters investigated were normal, and in both these patients also no differences were found in the naïve BCR repertoire. In the remaining 14 patients, we mainly found differences in SHM levels and subclass distribution (Supplementary Figure 3). The differences in the antigen selected repertoire is further visualized when performing PCA analysis based on all parameters analyzed in the IGHG transcripts (Figure 6B; Supplementary Figure 2B). As expected CVID5 clusters in between the controls (CVID6 is not included due to missing IGHG data) but all other patients cluster away from the controls to some extent indicating that IGHG repertoire of CVID patients is often altered in CVID patients.

## Discussion

CVID patients have a defect in antibody production often associated with a block or defect in B-cell development, but the stage or process effected varies. Here we investigated whether CVID patients have a defect in repertoire formation or specification by analyzing the naïve and antigen selected BCR repertoire. We mainly focused on deviating parameters in individual patients to identify disease causing or disease associated processes for individual patients instead of comparing CVID patients to HCs as done in previous studies (16, 17, 32, 33).

Using a patient-based approach we identified the GC response as the process in B-cell development that is most often affected in CVID. In the large majority of patients (*n* = 13) we find less usage of the distal constant domains which in eight patients is associated with a reduced level of SHM described before (17, 32, 34, 35). Earlier studies have already found reduced numbers of memory B-cells and/or plasmablasts in CVID suggesting a quantitative defect in B cell development in the GC (7, 14). We now show that in addition CVID patients have a qualitative defect in the cells exiting the GC. It would be interesting to further study the exact defect in the GC however, therefore it would be necessary to look in the secondary lymphoid organs of these patients and such material is rare.

We also looked in more detail at the patterns of SHM and identified five patients with minor alterations in their SHM patterns of which one would match a minor impairment in MMR. This is in line with earlier findings of Duvvuri et al. which found minor alterations in SHM patterns in some CVID patients and more extensive skewing in line with a MMR defect in one patient (33). The differences observed in our cohort are minor and unlikely to be the disease causing.

When analyzing markers indicative for antigen selection in IGHG and IGHA transcripts we did not observe any major deviation. In contrast, when analyzing selection against features associated with auto-immunity (IGHV4-34, CDR3 length, JH6 usage) we observed patients with increased auto-immune features in both the naïve and antigen selected repertoire. Most pronounced was the increased usage of IGHV4-34 in the naïve repertoire and IGHG transcripts. However, when studying the relation between the auto-immune repertoire features and clinical presentation we did not find a clear correlation. In part this might be explained by the fact that these repertoire features are associated with auto-immunity but are not causative. In addition, it might be that a subgroup of patients with increased auto-immune features will develop auto-immunity at a later time point.

When analyzing the naïve repertoire we found some patients with minor alterations which could be indicative of defects in early B-cell development or the initiation of V(D)J recombination. This is in line with our previous study on a subgroup of our cohort in which we found that the naïve repertoire is normal in the majority of CVID patients (16). In the present study we found a reduced repertoire diversity in four of the 27 CVID patients and minor alterations in the junctions (of productive sequences) in 11 patients. Importantly, we noticed that the diversity of the naïve BCR repertoire decreases with age in both the CVID patients and HC's (Figure 1A). It is therefore crucial that age-matched controls are used for the analysis of repertoire diversity. Roskin et al. have previously shown that on a population basis the diversity of the BCR repertoire was reduced in CVID patients. Unfortunately, in their analysis age was not taken into account making it difficult to identify if they also find individual patients with a reduced repertoire diversity in their cohort (17).

Although we find some alterations in the immune repertoire of almost all patients, in two patients the repertoire was completely normal (CVID5 and CVID6). This suggests that in these patients no qualitative defect is found in BCR repertoire formation or specification. Interestingly both patients were included in the study of Driessen et al. in which they were both categorized as CVID pattern 5 (7). This pattern identifies patients with impaired antibody production by plasma cells which is in line with our current findings.

In conclusion, in this study we analyzed the naïve and the antigen selected BCR repertoire in CVID patients to pinpoint the stage or process of B-cell development impaired in individual patients. By comparing the results of individual patients to HC's we were able to identify the GC reaction as the process most often deregulated in CVID patients. In addition, some patients have possible defects in early B-cell development or selection against auto-immune features. Together this indicates that in CVID, repertoire formation is intact in the majority of patients, while repertoire specification is often impaired (Figure 6C). This further highlights that CVID patients not only have a quantitative defect in B cell development, but that also the quality of B cells in these patients is impaired.

## Author contributions

PvS, HI, and MvdB designed research and wrote the paper. PvS, HI, IP-K, DvZ, and AS performed experiments and/or analyzed data. VD, PvH, and SP provided patient material, collected clinical data, and critically read the manuscript.

### Conflict of interest statement

The authors declare that the research was conducted in the absence of any commercial or financial relationships that could be construed as a potential conflict of interest.
