# Supplementary material for: Identification of CVID Patients With Defects in Immune Repertoire Formation or Specification
Source: Front Immunol. 2018 Nov 23;9:2545. doi: 10.3389/fimmu.2018.02545 (PMC6265514; doi:10.3389/fimmu.2018.02545)
Supplement: Supplementary file 1 [file Data_Sheet_1.docx]

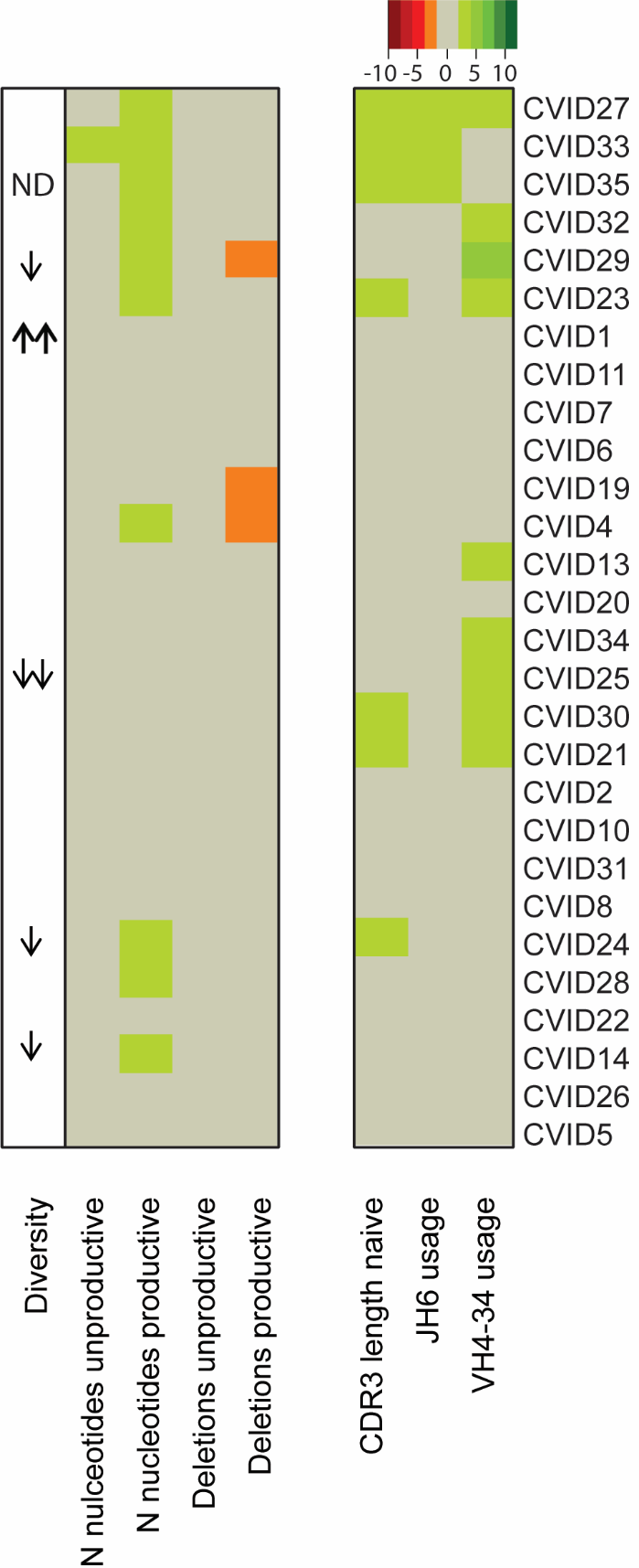


**Supplementary Fig. 1.** Heatmap including all data of the naïve BCR repertoire. Data are clustered based on all data depicted. Values which deviate >2 st dev from the mean are depicted in increasingly darker shades of red (decreased values) or green (increase values).


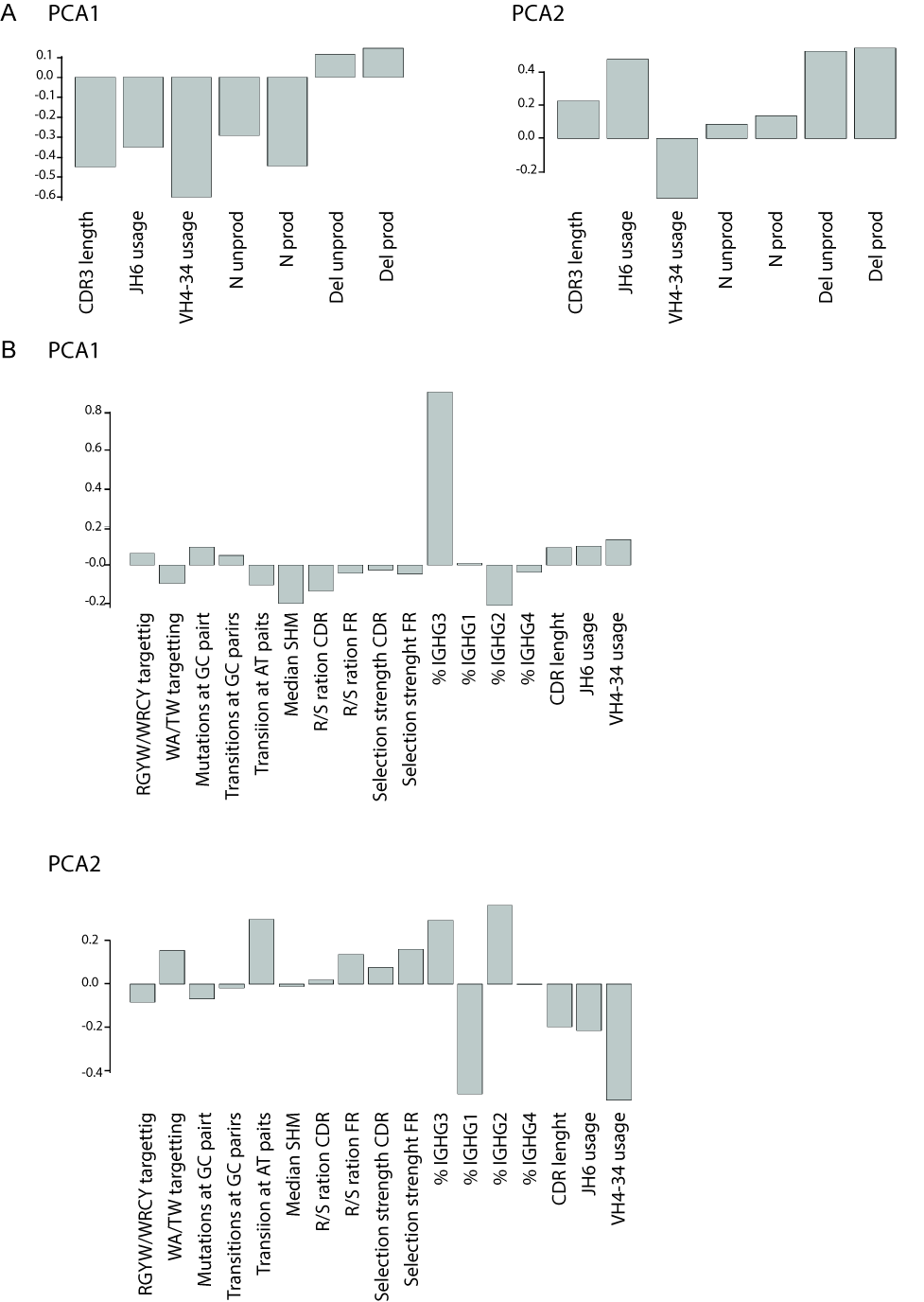


**Supplementary Fig. 2. Contribution of the different parameters to the PCA analysis. (A)** Contribution of the different parameters to PCA1 and PCA2 of the naïve repertoire. **(B)** Contribution of all parameters to the PCA analysis of the antigen selected repertoire.

**
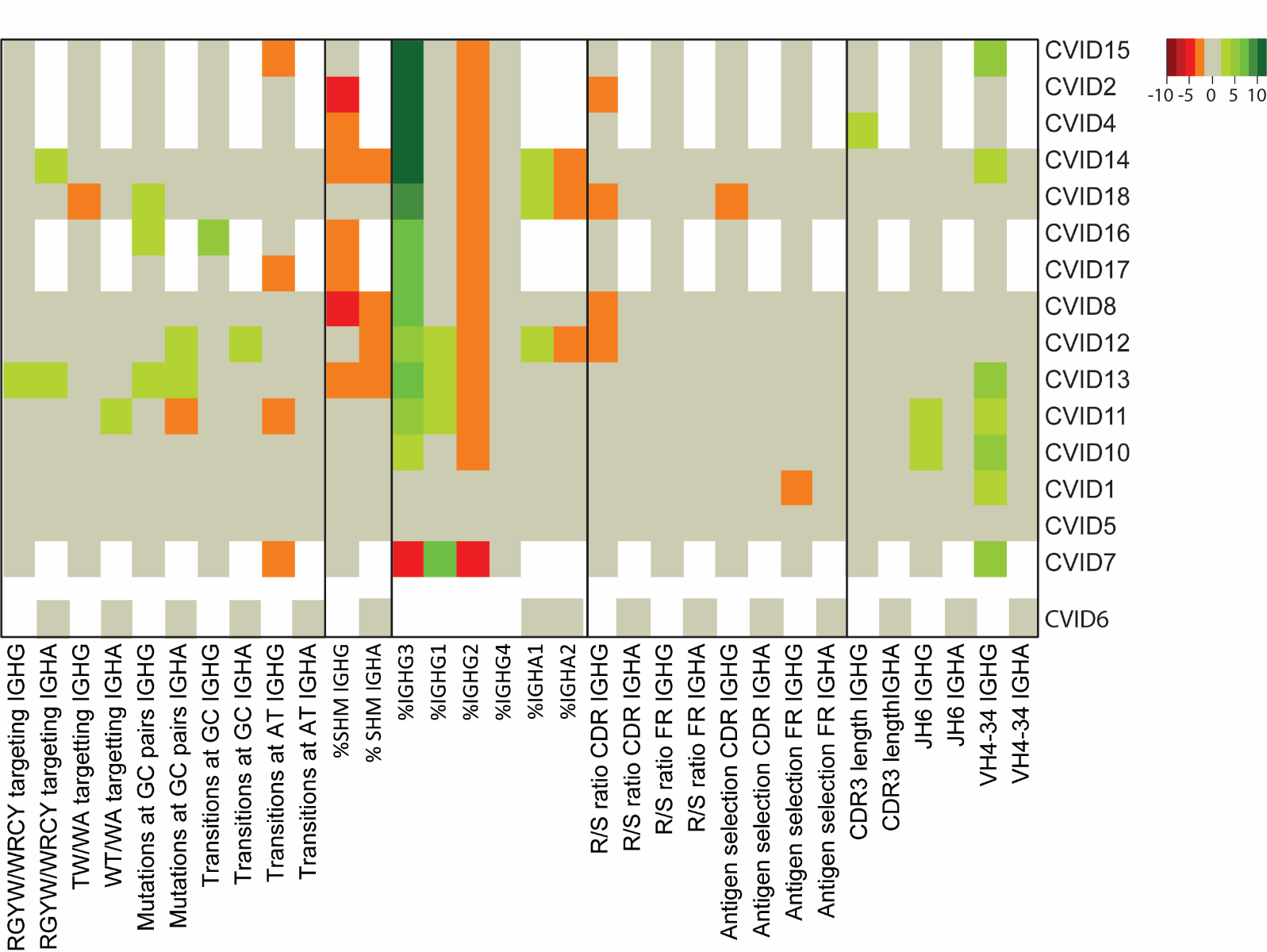
**

**Supplementary Fig. 3.** Heatmap showing all parameters analysed in the antigen selected BCR repertoire. Difference shades of red indicate values >2 st dev below the mean, while green indicates deviations >2 st dev above the mean. Patients are clustered based on all parameters analysed in the antigen selected BCR repertoire.

| Patient  Supplementary table 1: **Overview of patient characteristics.** Overview of all patients and all available immune phenotyping data including IgG, IgM and IgA levels at diagnosis. | Age (yr) | Sex | CD19 Count (x10*9/L) | Transitional B-cells Count (x10*6/L) | Naive mature B-cells Count (x10*6/L) | Marginal zone like B-cells Count (x10*6/L) | Memory B-cells Count (x10*6/L) | CD3 Count (x10*9/L) | CD4 Count (x10*9/L) | CD8 Count (x10*9/L) | IgG levels (g/L) | IgM levels (g/L) | IgA levels (g/L) | Auto-immunity |
| --- | --- | --- | --- | --- | --- | --- | --- | --- | --- | --- | --- | --- | --- | --- |
| Ref. values | 5-10 |  | 0.2-1.6 | 11-77 | 111-486 | 15-88 | 13-100 | 0.7-4.2 | 0.3-2.0 | 0.3-1.8 |  |  |  |  |
| Ref. values | 6-9 |  |  |  |  |  |  |  |  |  | 5,2-14,3 | 0,28-1,9 | 0,54-2,5 |  |
| CVID19 | 6 | Female | 0,64 | 9,5 | 497,55 | 45,75 | 26,05 | 2,06 | 1,37 | 0,44 | 0,13 | 0,37 | 2,33 |  |
| CVID20 | 7 | Male | 0,22 | 15,4 | 156,49 | 18,6 | 6,57 | 1,4 | 0,85 | 0,5 | 0,3 | 0 | 3,1 |  |
| CVID11 | 8 | Male | 0,34 | 8,3 | 244,08 | 31,7 | 18,07 | 1,37 | 0,69 | 0,42 | 4,2 | 0,36 | 0,42 |  |
| Ref. values | 10-16 |  | 0.2-0.6 | 4-108 | 87-390 | 7-90 | 10-76 | 0.8-3.5 | 0.4-2.1 | 0.2-1.2 |  |  |  |  |
| Ref. values | 12-16 |  |  |  |  |  |  |  |  |  | 5,2-15,6 | 0,13-1,6 | 0,62-3,0 |  |
| CVID7 | 12 | Female | 0,32 | 8,3 | 260,14 | 21,73 | 2,27 | 1,52 | 0,95 | 0,47 | 3 | 0,5 | 0,12 |  |
| CVID6 | 13 | Male | 0,34 | 10,3 | 261,35 | 20,343 | 10,34 | 1,91 | 1,12 | 0,71 | 4,2 | 0,64 | 0,23 |  |
| CVID21 | 15 | Female | 0,17 | 14,2 | 138,94 | 3,31 | 0,35 | 1,63 | 0,86 | 0,65 | 0,21 | 0,3 | 2 |  |
| Ref. values | Adults |  | 0.1-0.5 | 3-50 | 57-447 | 9-88 | 13-122 | 0.7-2.1 | 0.3-1.4 | 0.2-1.2 | 7,0-16 | 0,40-2,3 | 0,70-4,0 |  |
| CVID10 | 17 | Male | 0,25 | 4 | 207,37 | 8,88 | 8,63 | 1,26 | 0,76 | 0,38 | 4,49 | 0,36 | 0,48 |  |
| CVID22 | 17 | Male | 0,27 | 3,9 | 242 | 12,14 | 2,7 | 1,08 | 0,7 | 0,34 | 0,15 | 0,15 | 2,5 |  |
| CVID23 | 17 | Female | 0,02 | 25,9 | 15,62 | 0,56 | 0,14 | 0,43 | 0,26 | 0,12 | 0,03 | 0,22 | 0,6 |  |
| CVID8 | 18 | Female | 0,5 | 3 | 386,61 | 66,11 | 14,52 | 2,17 | 1,36 | 0,63 | 4,9 | 0,3 | 0,46 | Immune Thrombocytopenic Purpura and Autoimmune hemolytic anemia |
| CVID4 | 22 | Male | 0,13 | 5,9 | 111,66 | 6,95 | 1,29 | 0,86 | 0,41 | 0,35 | 3,1 | 0 | 0 |  |
| CVID24 | 22 | Male | 0,18 | 12,4 | 149,62 | 3,38 | 0,53 | 0,83 | 0,48 | 0,15 | 0,65 | 1,58 | 3,4 |  |
| CVID14 | 26 | Male | 0,42 |  |  |  |  | 1,34 | 0,91 | 0,4 | 3 | 0,2 | <0.1 |  |
| CVID17 | 32 | Male | 0,5 |  |  |  |  | 3,8 | 2,2 | 1,2 | 2,7 | 0,4 | 0,3 |  |
| CVID25 | 32 | Female | 0,28 |  |  |  |  | 0,83 | 0,64 | 0,19 | 3,8 | 0,4 | 0,1 |  |
| CVID26 | 34 | Male | 0,11 |  |  |  |  | 0,88 | 0,47 | 0,39 | 0,41 | <0.04 | <0.06 |  |
| CVID27 | 34 | Female | 0,2 |  |  |  |  | 0,91 | 0,46 | 0,35 | 2,7 | <0.04 |  | Autoimmunity, vitiligo |
| CVID29 | 40 | Female | 0,14 |  |  |  |  | 1,31 | 0,68 | 0,48 | 3,35 | 0,17 | 0,44 | Autoimmune cytopenias |
| CVID30 | 41 | male | 0,12 |  |  |  |  | 0,83 | 0,47 | 0,36 | 0,9 | 0,04 | <0.06 |  |
| CVID31 | 43 | Female | 0,34 |  |  |  |  | 0,86 | 0,54 | 0,27 | 3,07 | <0.07 | <0.06 |  |
| CVID2 | 44 | Female | 0,1 | 0,8 | 62,49 | 30,95 | 2,36 | 0,61 | 0,38 | 0,18 | 1,3 | 0,11 | 0 |  |
| CVID32 | 45 | Male | 0,15 |  |  |  |  | 1,84 | 0,79 | 1,13 | 0,25 | <0.04 | <0.06 |  |
| CVID5 | 46 | Female | 0,51 | 1,1 | 120,3 | 312,29 | 79,35 | 2,17 | 1,58 | 0,53 | 5,5 | 1,27 | 0 |  |
| CVID1 | 49 | Male | 0,14 | 5,7 | 86,64 | 24,91 | 13,14 | 0,88 | 0,71 | 0,12 | 5,7 | 0,31 | 0,59 |  |
| CVID12 | 52 | Male | 0,26 |  |  |  |  | 1,12 | 0,6 | 0,45 | 2,7 | 0,2 | <0.01 |  |
| CVID33 | 55 | Female | 0,14 |  |  |  |  | 0,51 | 0,25 | 0,27 | <2 | <0.3 | <0.1 |  |
| CVID15 | 58 | Male | 0,2 |  |  |  |  | 1,15 | 0,45 | 0,69 | 2,6 | 0,1 | <0.1 |  |
| CVID34 | 60 | male | 0,21 |  |  |  |  | 0,96 | 0,26 | 0,68 | 0,79 | <0.05 | <0.06 |  |
| CVID13 | 65 | Female | 0,26 |  |  |  |  | 1,18 | 0,82 | 0,43 | 2,6 | 1,3 | 0,1 |  |
| CVID35 | 65 | Female | 0,11 |  |  |  |  | 1,26 | 0,65 | 0,56 | 2.65 |  |  | Immune Thrombocytopenic Purpura |
| CVID16 | 75 | Female | 0,38 |  |  |  |  | 2,23 | 0,95 | 1,27 | 1,8 | 0,3 | <0.1 |  |
| CVID18 | 79 | Female | 0,2 |  |  |  |  | 1,66 | 0,59 | 1,11 | 0,2 | 0,2 | 0 | Autoimmune cytopenias |

| Supplementary table 2: **Overview of sample characteristics and unique IGH rearrangements.** Overview of all patients and HC samples. Codes of all patient samples start with CVID, while the HC start with NWK or perio. # sample excluded based on clonality; * sample excluded because low number of unique reads; ** samples excluded from calculation of diversity. |  |  | **antigen selected B cell repertoire** | | **naive B cell repertoire** | | | | | | |
| --- | --- | --- | --- | --- | --- | --- | --- | --- | --- | --- | --- |
| **sample** | **AGE** | **autoimmunity** | **IGHA** | **IGHG** | **replicate 1** | **replicate 2** | **replicate 3** | **replicate 4** | **replicate 5** | **replicate 6** | **sum** |
| CVID1 | 49 |  | 485 | 355 | 7564 | 8249 | 9026 | 5666 | 9255 | 6934 | 46694 |
| CVID2 | 44 |  | 1* | 322 | 1544 | 5319 | 4530 | 4457 | 6351 | 5572 | 27773 |
| CVID4 | 22 |  | 2* | 987 | 4768 | 3806 | 5507 | 4074 | 5199 | 4579 | 27933 |
| CVID5 | 46 |  | 374 | 167 | 2865 | 2966 | 2128 | 2148 | 3620 | 4960 | 18687 |
| CVID6 | 13 |  | 476 | 402# | 9125 | 10479 | 9622 | 8560 | 6173 | 7603 | 51562 |
| CVID7 | 12 |  | 113# | 143 | 9690 | 10979 | 9104 | 4952 | 5587 | 6657 | 46969 |
| CVID8 | 18 | yes | 1647 | 1930 | 1627 | 3425 | 4601 | 3752 | 4416 | 3504 | 21325 |
| CVID10 | 17 |  | 587 | 487 | 5906 | 4859 | 4734 | 7156 | 4299 | 5829 | 32783 |
| CVID11 | 8 |  | 160 | 196 | 7305 | 7376 | 6356 | 6048 | 8636 | 6930 | 42651 |
| CVID12 | 52 |  | 462 | 1003 |  |  |  |  |  |  |  |
| CVID13 | 65 |  | 657 | 531 | 13094 | 10599 | 3164 | 2370 | 3826 | 3589 | 36642 |
| CVID14 | 26 |  | 704 | 523 | 2460 | 2204 | 4366 | 3165 | 335 | 337 | 12867 |
| CVID15 | 58 |  | 33* | 431 |  |  |  |  |  |  |  |
| CVID16 | 75 |  |  | 113 |  |  |  |  |  |  |  |
| CVID17 | 32 |  |  | 933 |  |  |  |  |  |  |  |
| CVID18 | 79 | yes | 76 | 87 |  |  |  |  |  |  |  |
| CVID19 | 6 |  |  |  | 6949 | 7842 | 7890 | 8904 | 11314 | 9576 | 52475 |
| CVID20 | 7 |  |  |  | 8253 | 4732 | 4500 | 3758 | 2725 | 5768 | 29736 |
| CVID21 | 15 |  |  | 47# | 6615 | 8600 | 7832 | 7893 | 2408 | 5451 | 38799 |
| CVID22 | 17 |  | 223# | 86# | 4813 | 3033 | 3874 | 3058 | 4942 | 3416 | 23136 |
| CVID23 | 17 |  |  |  | 3443 | 2927 | 3402 | 2594 | 2314 | 3194 | 17874 |
| CVID24 | 22 |  | 52# | 42* | 3533 | 1983 | 1920 | 1030 | 2443 | 4926 | 15835 |
| CVID25 | 32 |  |  |  | 1493 | 2148 | 1908 | 2965 | 2719 | 2179 | 13412 |
| CVID26 | 34 |  |  | 35* | 7784 | 3441 | 3083 | 3970 | 471 |  | 18749 |
| CVID27 | 34 | yes |  |  | 2694 | 2084 | 6 | 2799 | 1539 |  | 9122 |
| CVID28 | 39 |  |  | 11* | 6413 | 6592 | 2923 | 1040 | 317 |  | 17285 |
| CVID29 | 40 | yes |  |  | 1127 | 2090 | 1865 | 3234 | 374 | 2629 | 11319 |
| CVID30 | 41 |  |  |  | 839 | 3931 | 3163 | 960 | 3712 |  | 12605 |
| CVID31 | 43 |  |  |  | 5403 | 2970 | 2980 | 3475 |  |  | 14828 |
| CVID32 | 45 |  |  |  | 2697 | 461 | 2106 | 1407 | 1311 |  | 7982 |
| CVID33 | 55 |  |  |  | 433 | 2051 | 742 | 2760 | 2568 | 869 | 9423 |
| CVID34 | 60 |  |  |  | 4369 | 4445 | 3468 | 3576 | 5363 | 3689 | 24910 |
| CVID35 | 65 | yes |  | 20* |  |  |  |  |  |  | 5989 |
| NWK3 | 2,5 |  |  |  | 6062 | 2826 | 1861 | 1025 | 986 | 819 | 13579 |
| NWK64 | 7 |  | 111 | 35* |  |  |  |  |  |  |  |
| NWK61 | 8 |  | 73 | 14* |  |  |  |  |  |  |  |
| NWK66 | 9 |  | 96 | 65 |  |  |  |  |  |  |  |
| NWK180 | 10 |  |  |  | 15042 | 3254 | 11113 |  |  |  | 29409 |
| NWK57 | 14 |  | 223 | 395 |  |  |  |  |  |  |  |
| NWK42 | 15 |  | 136 | 174 |  |  |  |  |  |  |  |
| NWK5 | 15 |  | 129 | 58 |  |  |  |  |  |  |  |
| NWK162 | 16,8 |  |  |  | 16055 | 12025 | 1992 |  |  |  | 30072 |
| NWK53 | 18 |  | 863 | 1063 |  |  |  |  |  |  |  |
| NWK43 | 20 |  | 277 | 316 |  |  |  |  |  |  |  |
| NWK302 | 20 |  |  |  | 11296 | 10160 | 6453 | 5369 | 4874 | 3652 | 41804 |
| NWK303 | 22 |  | ### | 914 |  |  |  |  |  |  |  |
| NWK290 | 24 |  |  |  | 7951 | 4217 | 5175 | 6104 | 5184 | 3505 | 32136 |
| NWK383 | 25 |  | 49 | 84 |  |  |  |  |  |  |  |
| NWK214 | 27 |  | 314 | 195 | 6514 | 4554 | 4506 | 4927 | 3831 | 4561 | 28893 |
| NWK297 | 31 |  | ### | 598 |  |  |  |  |  |  |  |
| NWK382 | 34 |  | 144 | 198 |  |  |  |  |  |  |  |
| NWK245 | 34 |  |  |  | 7671 | 5369 | 1650 | 2167 | 1646 | 10601 | 29104 |
| perio34 | 35 |  | ### | 784 |  |  |  |  |  |  |  |
| perio37 | 36 |  | 576 | 1469 |  |  |  |  |  |  |  |
| NWK237 | 37 |  | 905 | 494 | 3810 | 7530 | 8760 | 4869 | 6430 | 4264 | 35663 |
| NWK379 | 43 |  | 169 | 181 | 4138 | 3200 | 4385 | 4609 | 562 | 921 | 17815 |
| NWK380 | 43 |  | 27* | 92 | 5181 | 5346 | 3066 | 3564 | 1364 | 1376 | 19897 |
| NWK301 | 45 |  |  |  | 7927 | 7644 | 5866 | 6366 | 5476 | 4645 | 37924 |
| NWK299 | 47 |  | ### | 630 | 4990 | 5769 | 4439 | 5137 | 2526 | 3294 | 26155 |
| NWK378 | 49 |  | 354 | 540 |  |  |  |  |  |  |  |
| NWK377 | 55 |  | 917 | 469 | 1089 | 826 | 527 | 556 | 2646 | 1059 | 6703 |
| NWK384 | 56 |  | 35* | 41* | 761 | 685 | 999 | 325 | 1075 | 293 | 4138 |
| NWK385 | 60 |  | 75 | 26* | 7254 | 6069 | 4356 | 2124 | 3054 | 754 | 23611 |
| NWK381 | 67 |  | 88 | 126 | 1295 | 899 | 1969 | 1785 | 1946 | 1364 | 9258 |
| NWK387 | 72 |  | ### | 600 |  |  |  |  |  |  |  |
| NWK386 | 74 |  | ### | 839 |  |  |  |  |  |  |  |

**Supplemental Table 3. Overview of the number and type of mutations per sample**
